# The interplay of spiritual health, resilience, and happiness: an evaluation among a group of dental students at a state university in Turkey

**DOI:** 10.1186/s12903-024-04297-4

**Published:** 2024-05-21

**Authors:** Meltem Karahan, Bahar Basak Kiziltan Eliacik, Kursad Nuri Baydili

**Affiliations:** 1 Private Practice, Istanbul, Turkey; 2 Present Address: Department of Pediatric Dentistry, Hamidiye Faculty of Dental Medicine, University of Health Sciences, İstanbul, Turkey; 3 Present Address: Department of Biostatistics and Medical Informatics, Hamidiye Faculty of Medicine, University of Health Sciences, Istanbul, Turkey

**Keywords:** Spiritual health, Resilience, Happiness, Holistic health, Dentistry

## Abstract

**Background:**

Dental education is one of the disciplines where students are most significantly affected psychologically. The aim of this study was to evaluate the relationship between spiritual health, resilience and happiness levels of dental students at a state university in Turkey.

**Methods:**

This cross- sectional study included 212 students from the 3rd, 4th and 5th grades of the faculty of dentistry. A questionnaire consisting of 4 sections was used in the study. The sections of the questionnaire include students’ general and academic information, Turkish adaptations of the Spiritual Well-Being Scale, The Brief Resilience Scale, and the Oxford Happiness Questionnaire-Short Form. Data analysis was performed with IBM SPSS 25 package program. The Shapiro-Wilk test was used to assess the normal distribution of the data. The Mann-Whitney U test was preferred for comparisons between two categorical variables and one numerical variable. The Kruskal-Wallis H test was employed for comparisons involving two categorical variables and one numerical variable. The presence of a relationship between two numerical variables was examined using the Spearman test.

**Results:**

In terms of resilience and happiness scores, males had higher scores than females. It was determined that third graders scored higher than fifth graders in harmony with nature scores, and third graders scored higher than fourth graders in deregulation scores. There was a positive correlation between happiness, spiritual well-being and resilience; a negative correlation between happiness and anomie. There was no significant relationship between age and happiness scores. As a result of multiple linear regression to determine the factors affecting happiness; increases in spiritual well-being and resilience will lead to an increase in happiness levels.

**Conclusion:**

This study concluded that increased levels of spiritual well-being and resilience among a group of dental students would lead to increased levels of happiness. However, further research is needed to understand the relationship between mental health, resilience and happiness levels during dental education.

## Background

Mental health, acknowledged by the World Health Organization (WHO) as an integral dimension of health, encompasses a state of complete well-being, encompassing physical, mental, and social facets [1]. Recognizing mental health’s significance is pivotal as it not only facilitates comprehensive learning but also enhances overall physical and psychological well-being, thereby improving quality of life [2]. Dental education stands as one of the most demanding academic disciplines globally [3]. Given the role of dental students in safeguarding and promoting community health, maintaining high levels of mental well-being is essential, owing to the professional pressures inherent in their field [4].

Addressing stressors specific to dental education, including time constraints, internships, course loads, patient management, exam anxiety, and financial constraints, is crucial [5–7]. Furthermore, demographic factors such as age, gender, relationship status, personality type, and academic year have been linked to variations in stress levels among dental students [8–12]. Specifically, studies suggest higher stress levels among female dental students compared to their male counterparts [13–15]. Existing literature on medical students highlights the correlation between sufficient physical and psychological well-being and reduced levels of depression, stress, and burnout [16–18].

The state of spiritual well-being can be defined as a balanced state of openness towards spiritual development, encompassing the ability to communicate with others, finding meaning and purpose in life, and a sense of belief in and connection to a higher power [19]. Individuals determine their processes of understanding and living life in accordance with their own values and interpretations, encompassing personal, social, and transcendent aspects [20].

Spiritual well-being is analyzed across three sub-dimensions and their totality, encompassing commitment to transcendence, harmony with nature, and the meaning of life (anomie). Transcendence involves a connection to something beyond human self or relationships, such as cosmic power, transcendent reality, or God, incorporating beliefs, admiration, and reverence for the enigmatic source of the universe. Harmony with nature involves a sense of care, admiration, and curiosity towards the physical and biological aspects of existence. Anomie relates to individuals’ engagement with the meaning, purpose, and values of life, reflecting self-awareness regarding self-esteem and identity [20, 21]. In a study on the spiritual well-being of university students in the Asia-Pacific region, students with higher levels of spiritual well-being are less likely to experience depression, anxiety, and stress. All domains of spiritual well-being (transcendence, harmony with nature, and anomie) have been associated with lower levels of psychological distress [22].

Resilience can be conceptualized as the process through which individuals adapt to adversity and stress, constituting a fundamental element of well-being. Resilient individuals demonstrate healthy coping strategies and exhibit enhanced capacities to confront challenges [23]. Research indicates that resilient medical students exhibit lower rates of depression, enjoy a higher quality of life, demonstrate reduced likelihood of unemployment, encounter fewer stressful life events, report higher levels of social support, perceive their learning environment more positively, and experience lower levels of stress and fatigue compared to their vulnerable counterparts [18].

Happiness, construed as a mental or emotional state characterized by joy, life satisfaction, and the absence of negative emotions, is also intertwined with mental health [24]. Research indicating a positive correlation between spiritual health and happiness has also explored the relationship between resilience and happiness [25–27]. Evidence suggests a mutually reinforcing relationship among spiritual health, resilience, and happiness [28]. Considering the crucial role of spiritual health, resilience, and happiness in managing challenges faced by dental students, it is essential to examine their levels and interactions. The hypothesis of this study is that there is a positive relationship between spiritual well-being, psychological resilience, and happiness. Consequently, this study aims to assess the levels of mental health, resilience, and happiness among dental students and explore the mediating role of resilience in the relationship between mental health and happiness.

## Methods

### Study design

This cross- sectional study was conducted between June 2022 and August 2022 with the participation of 3rd, 4th, and 5th-year students enrolled in our university’s Faculty of Dentistry.

### Participants

#### Inclusion criteria

Students enrolled in the 3rd, 4th, and 5th years of the Faculty of Dentistry at Health Sciences University, who are willing to fill out the questionnaire and participate in the study voluntarily.

#### Exclusion criteria

Students enrolled in the 3rd, 4th, and 5th years of the Faculty of Dentistry at Health Sciences University, who are unwilling to fill out the questionnaire and participate in the study voluntarily, as well as students enrolled in the 1st and 2nd years of the Faculty of Dentistry at Health Sciences University.

Sample analysis was conducted as follows: The population of the study consists of students enrolled in the 3rd, 4th, and 5th years of the dentistry faculty. A complete enumeration method was employed for sample selection without resorting to sampling. The sample size for the study was determined as 328 individuals. A total of 212 students participated in the survey conducted within the specified dates.

### Ethical approval

For the study was obtained with decision number 16/17 from the University of Health Sciences Hamidiye Scientific Research Ethics Committee at the meeting held on 17.06.2022, recorded under registration number 22/360. This study performed in accordance with the Declaration of Helsinki.

### Intervention

After coordinating with class representatives, a web link leading to an anonymous questionnaire was distributed. The data collected from this questionnaire were solely used for academic purposes and were not shared with any external entities. Participants were duly informed with the following statement: “Providing responses to the questionnaire based on objective criteria will significantly contribute to the research.” Subsequent to obtaining ethical clearance, the questionnaires were dispatched by the principal investigator at biweekly intervals. The deadline for submitting the questionnaire was set as August 8, 2022. After this date, the questionnaire was closed for additional responses, and the collected data were carefully analyzed. Before starting the questionnaire, each participant provided clear and informed consent to take part in the study. Students from the Faculty of Dentistry who willingly agreed to fill out the questionnaire and join the research but either didn’t complete the questionnaire entirely or made mistakes in the reverse-coded questions were not included in the study group.

### Measurements

The questionnaire consisted of four sections. The first section included a total of three questions about the participants’ age, gender, and academic year.

#### Spiritual well-being scale

The second section was designed to assess the participants’ mental health. For this purpose, the Spiritual Well-Being Scale, adapted into Turkish by Ekşi and Kardaş in 2017 [29], was used. The spiritual well-being scale is a 29-item, 5-point Likert scale (1 = Not at all True to 5 = Completely True) developed for adults. It consists of three subscales: transcendence, harmony with nature, and anomie. In the analysis conducted by Halil Ekşi and Selami Kardaş, the structural validity and reliability of the scale have been demonstrated. Kaiser-Mayer-Olkin: 0.951, Eigen value was found to be 2, explaining 58.337% of the total item variance when Eigen value is set to 2; the model fit indices are as follows: (χ²/df = 4.11, Root-Mean-Square Error Approximation = 0.06, Standardized Root Mean Square Residual = 0.50, Normed Fit Index = 0.90, Comparative Fit Index = 0.92). It has been demonstrated that the scale has the ability to validly and reliably measure adults’ states of spiritual well-being [29].

#### Brief resilience scale

The third section was prepared to measure the participants’ resilience. For this purpose, the Brief Resilience Scale, adapted into Turkish by Tayfun Doğan in 2015 [30], was used. This scale is a 6-item self-report instrument using a 5-point Likert scale. Items 2, 4, and 6 are reverse-coded. After reversing the items, higher scores indicate higher spiritual resilience.

Adaptation of the Brief Resilience Scale into Turkish: A validity and reliability study conducted by Tayfun Doğan, the psychometric properties of the scale were examined through internal consistency, exploratory and confirmatory factor analysis, and criterion-related validity methods. The results of exploratory and confirmatory factor analysis indicated that the scale had a unidimensional structure. The internal consistency coefficient was found to be 0.83. In terms of criterion-related validity, positive correlations were found with several other scales. Consequently, it was concluded that the scale is a valid and reliable measurement tool for assessing psychological resilience in university students [30].

#### Oxford happiness questionnaire-short form

The fourth section was prepared to measure the participants’ happiness. For this purpose, the Oxford Happiness Questionnaire-Short Form, adapted into Turkish by Doğan and Çötok in 2011 [31], was used. It consists of 7 items, and there is a correlation of 0.93 (*p* < 0.001) between the short form and the original 29-item version. Participants are required to mark one option from the choices (1 = Strongly Disagree to 5 = Strongly Agree) for each item. Items 1 and 7 are reverse-coded. Higher scores indicate higher levels of happiness. The adaptation of the Short Form of the Oxford Happiness Questionnaire into Turkish, conducted by Tayfun Doğan and Nesrin Akıncı Çötok, employed Cronbach’s alpha for internal consistency and test-retest methods for reliability assessment. The Cronbach’s alpha coefficient for internal consistency was found to be 0.74. In the test-retest reliability study, a correlation of 0.85 was obtained. Based on these findings, it has been demonstrated that the reliability of the scale is at an acceptable level [31].

### Data analysis

Data analysis was performed using IBM SPSS 25 software. Frequency and percentage values were presented for categorical variables, and median, minimum, and maximum values were presented for numerical variables. The normality of the data was tested using the Shapiro-Wilk test. The Mann-Whitney U test was used for comparisons between two categorical variables and a numerical variable. The Kruskal-Wallis H test was used for comparisons between more than two categorical variables and a numerical variable. The presence of a relationship between two numerical variables was examined using the Spearman test. The Type I error rate was set at 0.05 in the study.

## Results

According to the gender of the participants; 39.6% (*n* = 84) were men and 60.4% (*n* = 128) were women. 48.6% (*n* = 103) are in the third grade, 39.2% (*n* = 83) are in the fourth grade, and 12.3% (*n* = 26) are in the fifth grade.

As a result of the comparisons between the genders in terms of scale and sub-dimension scores; It has been determined that men have higher scores than women in terms of resilience (*p* = 0.035) and happiness (*p* = 0.046) scores. There was no significant difference in terms of transcendence (*p* = 0.278), harmony with nature (*p* = 0.175), anomie (*p* = 0.477) and spiritual well-being scale total (*p* = 0.175) scores (Table 1).

**Table 1:** Comparisons between genders in terms of scale and sub-dimension scores

	Male	Female	Z	*p*
Transcendence	57 (15–75)	60 (15–75)	-1,085	0,278
Harmony with Nature	30 (9–35)	31 (14–35)	-1,355	0,175
Anomie	18 (7–35)	18,5 (9–35)	-0,712	0,477
Spiritual well-being Total	106,5 (51–142)	111,5 (43–142)	-1,358	0,175
Resilience	19 (10–30)	17 (6–30)	-2,11	0,035*
Happiness	21 (9–33)	19 (7–34)	-1,999	0,046*

*p < 0,05

As a result of the comparisons between the classes in terms of scale and sub-dimension scores; It was determined that third graders had higher scores than fifth graders in terms of harmony with nature scores (*p* = 0.044), and third graders had higher scores than fourth graders in terms of anomie scores (*p* = 0.011). Although it was determined that there was no significant difference in terms of transcendence (*p* = 0.772) and spiritual well-being scale total (*p* = 0.309) scores, there was a significant difference in terms of resilience (*p* = 0.045) and happiness (*p* = 0.036) scores in the Kruskal-Wallis H test. It was concluded that there was no significant difference in the comparisons between the categories (Table 2).

**Table 2:** Comparisons between grades in terms of scale and sub-dimension scores

	3	4	5	H	*p*	Differences
Transcendence	58 (15–75)	60 (15–75)	55,5 (19–74)	0,517	0,772	-
Harmony with Nature	31 (9–35)	30 (14–35)	28 (11–35)	6,247	0,044*	3 > 5
Anomie	20 (9–35)	17 (9–33)	20 (7–27)	9,031	0,011*	3 > 4
Spiritual well-being Total	111 (52–142)	112 (43–142)	101,5 (51–141)	2,351	0,309	-
Resilience	17 (6–30)	18 (6–30)	20,5 (12–30)	6,195	0,045*	-
Happiness	19 (9–33)	22 (7–34)	18,5 (11–33)	6,627	0,036*	-

As a result of examining the existence of a relationship between quantitative variables; There was no significant relationship between age and transcendence (*p* = 0.176), anomie (*p* = 0.805), spiritual well-being scale total (*p* = 0.133), resilience (*p* = 0.651) and happiness (*p* = 0.658) scores; It was determined that there was a very weak negative correlation between age and harmony with nature scores (*p* = 0.041; *r*=-0.140). There is a weak positive relationship between transcendence and harmony with nature (*p* < 0.001; *r* = 0.376), a very weak negative relationship between transcendence and anomie (*p* = 0.012; *r*=-0.173), a positive relationship between transcendence and spiritual well-being scale total scores. It was found that there was a perfect positive relationship (*p* < 0.001; *r* = 0.921) and a weak positive relationship between transcendence and happiness (*p* < 0.001; *r* = 0.317), while there was no significant relationship between transcendence and resilience (*p* = 0.868). There was a weak negative relationship between the scores of harmony with nature and anomie (*p* = 0.001; *r*=-0.234), a moderate positive relationship between the scores of harmony with nature and spiritual well-being scale total scores (*p* < 0.001; *r* = 0.536), with nature It was determined that there was a very weak positive correlation between adjustment scores and resilience (*p* = 0.049; *r* = 0.136), and a weak positive correlation between harmony with nature scores and happiness scores (*p* < 0.001; *r* = 0.316). There is a moderate negative correlation between anomie and spiritual well-being total scores (*p* < 0.001; *r*=-0.454), a high negative correlation between anomie and resilience (*p* < 0.001; *r*=-0.631), and a negative relationship between anomie and happiness. It was determined that there was a high directional relationship (*p* < 0.001; *r*=-0.642). There is a very weak positive correlation between spiritual well-being scale total scores and resilience (*p* = 0.004; *r* = 0.196) and a moderate positive correlation between spiritual well-being scale total scores and happiness (*p* < 0.001; *r* = 0.471) has been found to be. It was determined that there was a moderate positive correlation between resilience and happiness (*p* < 0.001; *r* = 0.555) (Table 3).

**Table 3:** Examination of the existence of a relationship between quantitative variables

	Correlation	Age	Transcendence	Harmony with Nature	Anomie	Spiritual well-being Total	Resilience
Transcendence	r	-0,093	-	-	-	-	-
	p	0,176	-	-	-	-	-
Harmony with Nature	r	-0,140	0,376	-	-	-	
	p	0,041*	< 0,001*	-	-	-	-
anomie	r	-0,017	-0,173	-0,234	-	-	-
	p	0,805	0,012*	0,001*	-	-	-
Spiritual well-being Total	r	-0,103	0,921*	0,536*	-0,454*	-	-
	p	0,133	< 0,001*	< 0,001*	< 0,001*	-	-
Resilience	r	0,031	0,011	0,136*	-0,631*	0,196*	-
	p	0,651	0,868	0,049*	< 0,001*	0,004*	-
Happiness	r	-0,031	0,317	0,316	-0,642	0,471	0,555
	p	0,658	< 0,001*	< 0,001*	< 0,001*	< 0,001*	< 0,001*

*p < 0,05

As a result of the multiple linear regression performed to determine the factors affecting happiness; It has been determined that increases in spiritual well-being (*p* < 0.001) and resilience (*p* < 0.001) will cause an increase in happiness levels. It was determined that age values did not affect the level of happiness (*p* = 0.683) (Table 4).

**Table 4:** Determination of factors affecting happiness

	B	Std.deviation	Beta	t	*p*	%95 CI
(Constant)	1,49	4,848		0,307	0,759	-8,067 − 11,047
Age	-0,082	0,2	-0,021	-0,41	0,683	-0,476-0,312
Spiritual well-being	0,096	0,014	0,358	6,96	< 0,001*	0,069 − 0,123
Resilience	0,583	0,057	0,526	10,27	< 0,001*	0,471-0,694

*p < 0,05 | R^2^ = 0,475 | model *p* < 0,001

## Discussion

Men and women exhibit differences not only in biological aspects but also in brain activity, gender-specific cognitive and behavioral styles, as well as susceptibility to diseases and disorders [32, 33]. Research on gender disparities underscores the importance of considering both genetic/hormonal and social influences [34]. A study on resilience during the Covid-19 pandemic found that men tended to exhibit higher levels of resilience than women [35]. Similarly, research conducted in Pakistan indicated that male university students reported higher levels of happiness than females [36]. In the present study, comparisons of scale and subscale scores between genders revealed that men exhibited higher scores in terms of resilience and happiness compared to women. This study uncovered a fascinating revelation: men are more resilient and happier compared to women.

Spiritual well-being enhances individuals’ self-esteem and promotes a positive perspective on life by helping them build meaningful connections with their surroundings [37, 38]. Hence, individuals who are mentally healthy possess the capacity to cultivate robust, positive relationships with others and their surroundings. Individuals derive existential significance from their affiliation with divine entities or higher objectives, concurrently exhibiting a cognizance of adverse emotions and cognitions intertwined with their life occurrences [29, 37]. Transcendence denotes the recognition of a higher power or entity beyond oneself, while harmony with nature pertains to an individual’s ability to adapt to and coexist harmoniously with nature and the environment. Furthermore, anomie signifies negative reactions experienced in life situations, often stemming from a sense of disconnection or lack of social cohesion [29].

According to a study conducted by Gencer et al. among university students, women achieved higher total scores in the sub-dimensions of transcendence and harmony with nature compared to men. Conversely, men obtained higher average scores in the sub-dimension of anomie [39]. Another study, as identified by Sen et al., reported significantly higher average scores in mental health for female students compared to male students [2]. However, in the current study, no significant differences were observed in terms of transcendence, harmony with nature, anomie, and overall spiritual well-being scale scores between male and female students. Similar levels of spiritual well-being were observed in both genders.

Ziapour et al. conducted a study investigating the influence of demographic variables on spiritual well-being among medical, dentistry, and pharmacy students in Iran. The findings indicated that students in the second grade exhibited the highest mean and standard deviations of spiritual well-being, while those in the fourth grade showed the lowest. However, no statistically significant difference was observed in the average scores of spiritual health among students across different grades [40]. In this study, comparisons among different grade levels indicated that third-grade students exhibited higher scores in harmony with nature compared to fifth-grade students. Additionally, third-grade students displayed higher scores in anomie compared to fourth-grade students. However, no significant differences were found in transcendence and overall spiritual well-being scale scores across the various grade levels. While spiritual well-being appeared similar across all grades, third-grade students exhibited notable differences in the subgroups of harmony with nature and anomie.

Happiness is defined as an individual’s assessment of their overall quality of life, which incorporates both positive and negative emotions, based on their internal standards [41]. On the other hand, resilience refers to an individual’s capacity to adapt effectively in the presence of challenging circumstances. It’s important to know that happiness and resilience are closely connected to an individual’s physical and mental well-being [40]. A study conducted among university students showed a positive correlation between happiness and resilience [42]. In this study, although significant differences were found in resilience and happiness scores, no significant differences were found in the comparisons between categories.

Spiritual crises are frequently encountered, especially among older children, wherein hope and faith often act as protective factors, fostering resilience and preserving the sanctity of life. An investigation focusing on young individuals diagnosed with cancer from a pediatric oncology clinic unveiled that the 15–17 age cohort demonstrated elevated levels of spiritual well-being in comparison to the 18–20 age [43]. A study investigating the correlation between emotional intelligence and happiness in medical students found a significant negative association between age and happiness, indicating a decline in happiness with increasing age [44]. Morgan et al. reported a consistent decline in happiness associated with aging in developing countries, which aligns with the findings of the present study [45].

Schiebe et al. conducted a study exploring the correlation between resilience and age among individuals engaged in remote work during the COVID-19 pandemic. Their findings revealed a pattern of relatively low resilience in the youngest age group, with a gradual increase observed in older age groups [46]. However, the study did not find a significant relationship between age and spiritual well-being, resilience, and happiness. Age did not emerge as a significant factor influencing spiritual well-being, resilience, or happiness.

A study conducted among individuals diagnosed with psoriasis revealed a positive and statistically significant relationship between spiritual well-being and resilience [47]. Another study, focusing on a cohort of elderly individuals, elucidated that those with moderate levels of spiritual well-being demonstrated a reduction in happiness levels [48]. In this investigation, a marginal positive relationship was identified between the total scores of the spiritual well-being scale and resilience, whereas a moderately positive relationship was observed between the total scores of the spiritual well-being scale and happiness. The study uncovered an interesting relationship: the spiritual well-being scale showed a slight link with resilience and a stronger one with happiness.

In a comparative study involving third-year medical and dentistry students, found that individuals with high resilience reported elevated levels of happiness and life satisfaction [27]. These findings underscore the significance of resilience in promoting well-being among medical and dental students. Resilience and happiness are closely associated.

Botor et al. conducted a study among adolescents in the Philippines, revealing a positive association between hope, personal well-being, resilience, and happiness [49]. Similarly, a study among dentistry students found a correlation between heightened levels of happiness, resilience, and increased spiritual health [28]. Moreover, the latter study revealed a positive correlation between increased spiritual well-being and resilience levels and heightened happiness levels. These results highlight the interrelation of spiritual health, resilience, and happiness across various demographics, including adolescents and dental students.

In this study, the sample was restricted to third, fourth, and fifth-year students enrolled at the Hamidiye Dental Faculty of the Health Sciences University. Limiting the sample to this specific subset of dental students in Turkey represents one of the study’s limitations.

Moreover, there is a paucity of research in the existing literature investigating the interplay between spiritual health, resilience, and happiness. Additionally, this study did not account for other potential variables influencing mental health, such as academic burnout. Therefore, further research is warranted to explore the impact of additional variables on spiritual health and its relationship with resilience and happiness.

## Conclusion

In conclusion, the journey of dental students is marked by significant academic stress, both in theory and practice, setting them apart from peers in other fields. Maintaining their spiritual well-being, resilience, and happiness is crucial during this demanding period. This study indicates a positive correlation between increased spiritual well-being and resilience levels among dental students and elevated happiness levels. However, further research is needed to explore the complex interplay among spiritual health, resilience, and happiness within the specific context of dental education. Understanding these dynamics more deeply can inform strategies to support the holistic well-being of dental students and enhance their overall academic experience.

Adaptation of various techniques such as stress management or emotional intelligence exercises to academic education is necessary to enhance levels of spiritual well-being, psychological resilience, and happiness among dental students. The integration of these methods into the academic training of dental students can enhance their spiritual well-being, strengthen their psychological resilience, and facilitate a happier student experience. Through the implementation of these strategies, a positive impact can be exerted on both the academic achievements and the overall quality of life of students.

## Data Availability

The data that support the findings of this study are available from the corresponding author, [Meltem KARAHAN], upon reasonable request.
